# Diagnosing diagnoses – can we improve our taxonomy?

**DOI:** 10.3897/zookeys.1071.72904

**Published:** 2021-11-16

**Authors:** Art Borkent

**Affiliations:** 1 691-8th Ave. SE, Salmon Arm, British Columbia, V1E 2C2, Canada unaffiliated Salmon Arm Canada

**Keywords:** Taxonomy, keys, identification

## Abstract

Taxonomic diagnoses should be clear but minimal statements that precisely distinguish a given specimen from other taxa at the same stage of development (e.g., pupa, adult female, egg). Presently, most diagnoses are of uncertain value. It is a great advantage for readers to be able to simply and confidently confirm their identifications after using a key.

There are numerous features that are important components of systematic treatments. The description of species, a functional key, portrayal of distributions, and discussion of associated taxonomic issues are standard in such publications. Additionally, many authors provide a diagnosis of the taxon at hand. These diagnoses, however, strongly vary in what is included.

In most publications during the past decades, diagnoses are often, at least within literature dealing with Diptera, a set of features that an author deems valuable or interesting in portraying a given taxon. Often, they are a summary of various character states without any specific purpose or only some of which distinguish the taxon. Whether authors desire to include such a summary or not, many diagnoses are not diagnostic, at least as defined by the Oxford dictionary: “the distinctive characterization in precise terms of a genus, species, or phenomenon” [one of two definitions]. Ernst Mayr (1969) in his book ‘Principles of Systematic Zoology’ defines a diagnosis as “in taxonomy, a formal statement of the characters (or most important characters) which distinguish a taxon from other similar or closely related coordinate taxa”. In ‘Phylogenetics, the Theory and Practice of Phylogenetic Systematics’, Wiley (1981) states that a diagnosis is “a brief listing of those characters which differentiate a taxon from related and/or similar taxa”. In the English glossary of the International Code of Zoological Nomenclature (4^th^ edition) a diagnosis is “A statement in words that purports to give those characters which differentiate the taxon from other taxa with which it is likely to be confused.” Dubois (2017) provides a more restricted understanding of the use of diagnoses, noting “the most widespread understanding of the term ‘diagnosis’ in taxonomy can be put as ‘list of taxonomic criteria allowing one to distinguish two different taxa’ when the latter are compared”. A comparison of only two taxa is often insufficient in groups with many taxa.

Rather than being a mix of character states of uncertain value in recognizing a taxon, it would therefore be a valuable contribution to every taxonomic paper to include a definitive diagnosis that allows a reader to confirm, in the simplest manner, the identification of a specimen at hand (after perhaps running it through a key). If there are further diagnostic features, the author can easily state that the taxon is unique in possessing character states 1+2+3 or character states 2+3+4, etc.

Brown et al. (2009, 2011) presents a comprehensive compendium allowing for the identification of all genera of Central America Diptera in a family-by-family treatment. Each family chapter provides a purported diagnosis, but the purpose of such diagnoses is unclear. The Culicidae (mosquitoes), for example, has the following lengthy diagnosis of the adult stage: “Adults slender (Fig. 1), 3–8 mm long (from anterior margin of clypeus to end of abdomen), 1–2 mm high (from upper margin of scutum to base of coxae). Head small, ovoid. Ocelli absent. Eye reniform, occupying most of side of head. Antenna with short, ringlike scape, enlarged globular pedicel, and 13-flagellomeres, usually more plumose in male. Proboscis long, slender, external part (labium) covered with scales. Thorax with patches of scales, as well as patches or rows of setae; setae usually coalesced on scutum into three paired, longitudinal rows: acrostical, dorsocentral, and supra-alar setae. Wing elongate, rounded apically, with scales along length of veins, microtrichia on membrane. Abdomen 10 segmented, segments 1–9 at least partly covered with scales in Culicinae bare in Anophelinae.” It is unclear whether the reader needs to check each of these features to be certain of the family identification of a specimen run through the family key. In fact, all extant adult Culicidae can be recognized by checking only two character states: an elongate proboscis, equal or longer than the antenna, and the presence of scales on the wing. These two features in combination are diagnostic within the order. If the reader had this knowledge, she/he could easily confirm the identification of the specimen being studied. In this instance, both sexes can be recognized using these features. Further to this, in each diagnosis, it should be clear what semaphoront (life stage) is being discussed, so that in this case the statement, “Male and female:” should precede the diagnostic features. If male and female features are otherwise both included in a single diagnosis, as in “Male with curved parameres, female with spherical spermatheca”, it would actually mean that features of both sexes are required for confirmation of the identification. As such, males and females generally need to be diagnosed separately, especially at the species level.

Diagnoses need to be restricted to the group under study. As such, the diagnosis of a given species in a generic study need only supply those features that are a unique combination within that genus. To be clear, a statement indicating the group considered should be provided, as in the example of *Corethrella* Coquillett species below. If authors provided such accurate diagnoses, students of our group would be more confident in identifying at every level of classification. They would clearly know, as they studied the literature, that an adult insect they collected in the Nearctic was a Diptera (the only order of insect worldwide with metathoracic halters), a Chaoboridae (the only family of Diptera worldwide with scales on the posterior margin of the wing, mouthparts shorter than the antenna, and wing vein R_1_ extending to near the apex of R_2_), a *Mochlonyx* Loew (the only Nearctic genus of Chaoboridae with the first tarsomere of each leg shorter than the second), and *Mochlonyxcinctipes* (Coquillett) (the only species of *Mochlonyx* in the Holarctic region with patterned wings).

In a revision of the genus *Corethrella* (Borkent 2008), a diagnosis for each of the 97 extant species was provided. In some instances, males and females could be diagnosed together because the unique set of features was present in both sexes. *Corethrellanippon* Miyagi was diagnosed as follows: “*Male and female adults*: only extant Old World species of *Corethrella* with a plain wing (no pattern of pigmentation), the scutum paler than the dark brown pleura, and the base of the hind tibia without pigmentation (equal to the apex of the hind femur).” In other species the males and females could not be diagnosed together and therefore were distinguished as in the following example of *Corethrellablandafemur* Borkent: “*Male adult*: only extant species of *Corethrella* with a stout, elongate, and apically expanded bristle on flagellomere 6. *Female adult*: only extant species of *Corethrella* in the New World with a circular head (in anterior view), with flagellomere 1 moderately elongate, sensilla coeloconica present only on flagellomeres 1, 9–13 and with only a single sensillum coeloconicum on each of 9–13, wing with only setae, with uniformly pigmented wing, scutum, katepisternum (with or without a very narrow dorsal pale band), and legs.” Supportive illustrations were provided and cited in the original diagnoses so that the reader can easily check features.

In many publications, systematic treatments are regional, or knowledge is more limited, and authors therefore may need to modify their diagnoses within a regional context, as in the *Corethrella* examples above, where identification of *Corethrellablandafemur* depends in part on where the features are considered distinctive (i.e., in the New World). If regional treatments can be sure of features being unique in a broader area, this should be stated as such: a Nearctic generic treatment should, if the author can present this, provide the features of a species as being unique worldwide. If restricted to the Nearctic, it would present the possibility to the reader that it may not be distinguishable using those character states from a Palaearctic species or an invasive from elsewhere.

Regardless whether the reader agrees with the statements above or not regarding diagnoses, there remains a need to help the users of our taxonomic work to confirm identifications as easily as possible. As taxonomists we want our work to be as clear and useful as possible. The keys we write are not for ourselves but for others who follow and who are uncertain of identifications (or they would no be using the key in the first place). When keying material of unfamiliar groups, it is a nearly universal emotion to feel some level of uncertainty in coming to a particular name. We all wish the author of the key could confirm the specimen identification we have determined. In the absence of teleporting, a diagnosis is the author’s opportunity to provide such affirmation. This is especially true in cases where keys are long and character states finely defined.

One reviewer pointed out that a diagnosis may hide the presence of further new species and that adding numbers of character states in a diagnosis helps the reader to avoid this. However, it appears to me that the opposite is true. If another researcher recognizes two taxa which both share a single published diagnosis, it provides clear evidence that one of the species is undescribed (or previously unknown from the area if the published study is restricted geographically). Otherwise, a reader who wants to examine other character states of a species can turn to the description for further details.

Some may argue that dichotomous keys provide the diagnostic features for a given taxon and although true, it is mostly a more complicated set of character states that needs to be considered. Taking the example of the Culicidae from above, this family keys out to one of the alternatives in couplet 8 in the family key in the Manual of Central American and couplet 15 of the Manual of Nearctic Diptera. For both, a number of other features need to be examined to arrive at this family. It is true that some diagnoses, with the minimum number of features allowing identification, are actually a sum of the features present in the key. However, in such instances (the minority) it is useful for the reader to know that all the features, already presented in the key, need to be checked for confirmation.

The increasing use of DNA barcodes has paved the way for describing new species characterized by a sequence shown or believed to be unique, and in some cases devoid of morphologically based diagnoses (e.g., Sharkey et al. 2021). For some, this is a panacea to deal with the often-overwhelming diversity present in some tropical habitats and/or hyperdiverse genera noted for small or miniscule morphological differences. There are, however, serious challenges that indicate the questionable interpretation of such results (Ahrens et al. 2021; Meier et al. 2021). What remains, however, is that there are currently some groups of species which are so morphologically similar that it is not possible to either key them and, by extension, provide a diagnosis. The evidence for treating them as species may be entirely behavioral or genetic (so that the diagnosis can only be a sequence). Further to this, it is clear that some life stages may not be diagnosable (e.g., the eggs of many species), with the evidence for treating them as separate species for some being present in only one stage. In such instances, it is most clear to state this in the diagnosis section of the systematic treatment (e.g., “Female adult not diagnosable to species”). Of course, future research may discover character states that do allow diagnosis of a given life stage. Regardless, such statements can make it clear to the reader as to which semaphoronts can or cannot be identified.

I have not, in this paper, compiled statistics on how many systematic treatments provide accurate diagnoses. However, experience with a few large systematic projects in Dipterology (the study of flies), reviewing more than 30 manuscripts per year for several decades (mostly taxonomic), and counseling students in their systematic projects, I diagnose a strong majority of diagnoses either to not to be diagnostic at all or to have diagnostic features included among a much larger array of character states. Further to this, among both students and colleagues, I have repeatedly encountered differences in opinion regarding the nature of diagnoses of species, genera, and other taxa. It would be beneficial, in my opinion, to re-examine our concepts of diagnoses and perhaps refine our presentation of this aspect of our taxonomic publications. I would also encourage editors of systematic papers to introduce more rigor in what is expected in a diagnosis for submitted papers.
